# Effects of memantine on mania‐like phenotypes exhibited by *Drosophila Shaker* mutants

**DOI:** 10.1111/cns.14145

**Published:** 2023-03-21

**Authors:** Ignazia Mocci, Maria Antonietta Casu, Valeria Sogos, Anna Liscia, Rossella Angius, Francesca Cadeddu, Maura Fanti, Patrizia Muroni, Giuseppe Talani, Andrea Diana, Maria Collu, Maria Dolores Setzu

**Affiliations:** ^1^ Institute of Translational Pharmacology National Research Council, Science and Technology Park of Sardinia Cagliari Italy; ^2^ Department of Biomedical Sciences University of Cagliari Monserrato Italy; ^3^ Unit of Biomedical Research Support, NMR Laboratory and Bioanalytical Technologies Sardegna Ricerche, Science and Technology Park of Sardinia Cagliari Italy; ^4^ Institute of Neuroscience National Research Council Monserrato Italy

**Keywords:** bipolar disorder, *Drosophila*, glutamate, memantine, NMDA receptor, *Shaker channel*

## Abstract

**Introduction:**

Increased glutamate levels and electrolytic fluctuations have been observed in acutely manic patients. Despite some efficacy of the non‐competitive NMDA receptor antagonist memantine (Mem), such as antidepressant‐like and mood‐stabilizer drugs in clinical studies, its specific mechanisms of action are still uncertain. The present study aims to better characterize the *Drosophila melanogaster* fly *Shaker* mutants (SH), as a translational model of manic episodes within bipolar disorder in humans, and to investigate the potential anti‐manic properties of Mem.

**Methods and Results:**

Our findings showed typical behavioral abnormalities in SH, which mirrored with the overexpression of NMDAR‐NR1 protein subunit, matched well to glutamate up‐regulation. Such molecular features were associated to a significant reduction of SH brain volume in comparison to Wild Type strain flies (WT). Here we report on the ability of Mem treatment to ameliorate behavioral aberrations of SH (similar to that of Lithium), and its ability to reduce NMDAR‐NR1 over‐expression.

**Conclusions:**

Our results show the involvement of the glutamatergic system in the SH, given the interaction between the *Shaker* channel and the NMDA receptor, suggesting this model as a promising tool for studying the neurobiology of bipolar disorders. Moreover, our results show Mem as a potential disease‐modifying therapy, providing insight on new mechanisms of action.

## Graphical abstract

Amelioration of functional and metabolic parameters in *Drosophila* mutant upon memantine treatment.

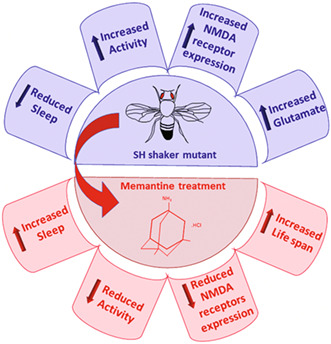

## 1 INTRODUCTION

Abundant experimental evidence supports the hypothesis that dysfunction of the NMDA glutamate receptors (NMDAR) and the dysregulation of their trafficking, can contribute to different neuropsychiatric disorders^1^, ^2^ including Bipolar Disorder (BD).^3^ BD is a severe psychiatric disorder characterized by episodes ranging from depression to euphoria.^4^ The heterogeneity of BD manifestation is expressed by the attempt to identify patient subtypes, based on the assessment of the overall brain morphology, brain region activation, functional connectivity, and white‐matter integrity.^5^ This complexity has propelled pharmacological research toward a highly inclusive drug design, aimed at targeting therapeutical hallmarks.^6^

Its pathogenesis is still unknown, but consistent literature suggests the presence of an underlying ionic homeostasis unbalance. Specifically, bipolar patients appear to have elevated intracellular sodium (Na^+^) levels, and free intracellular calcium (Ca^++^) concentrations, as well as reduced Na^+^ and potassium‐activated adenosine triphosphatase (Na^+^/K^+^‐ATPase), or Na^+^ pump activity in erythrocytes.^7^ The same phenomenon has been replicated in cortical neurons of mutant mice as valuable models of mania.^8^ Moreover, several clinical studies have strongly suggested a crucial role of altered glutamate levels and metabolism in the pathophysiology of BDs,^9^, ^10^, ^11^ although the specific mechanism by which this neurotransmitter boosts such disorders is still unclear. So far, clinical studies have demonstrated the efficacy of the non‐competitive NMDAR antagonist Memantine (Mem), which is extensively used for Alzheimer's disease therapy^12^ as an antimanic and as a mood‐stabilizing agent in Lithium (Li^+^) resistant patients affected by BD.^13^

Bipolar disorder features intermittent episodes of mania and depression, and several animal models attempt to recapitulate both components of this disorder.^14^

Symptoms of human mania include: increased motor activity, irritability, reduced need for sleep or changes in sleep patterns, aggressive behavior, increased sexual drive, distractibility, and risk‐taking behavior.^15^ However, since there are very few mammalian models that recapitulate all the human manic symptoms, *Drosophila melanogaster* (*Dm*) is a model organism owing to some specific advantages has also been used to study psychiatric disorders such as autism or schizophrenia^16^ and the mechanism of drug action. Thus, this model could lead to a better understanding of the BD pathogenesis aiming to disclose, and/or deepen, knowledge in the mechanisms of action.

Previous reports have shown motor hyperactivity and sleep disorders in the Drosophila model of neurodegenerative diseases, due to a possible dysregulation of some potassium channels.^17^

Hyper‐excitability, motor hyperactivity, and reduced sleep are also present in some *Dm* strains, such as the SH mutants, where such a gene (*SH*), located on the X chromosome, has its vertebrate counterpart in Kv1.1 gene.^18^

The resultant channel, named *Shaker*, is voltage‐dependent with four subunits forming a pore, allowing the flow of ions, which carry a type‐A potassium current.^19^ In *Dm* SH, a point mutation is present in the alpha‐subunit of this tetrameric voltage‐dependent potassium channel, and consequently, responsible for K^+^ currents attenuation. Since such current is involved in repolarizing action potentials (APs), SH flies show less repolarization of APs. The mutation in the *SH* gene reduces the K^+^ conductance in neurons, causing severe phenotypical aberrations in humans,^20^ with behavioral alterations expressed by the mini sleep phenotype and their shortened lifespan.^18^, ^19^, ^20^, ^21^ Notably, disturbances in sleep/wake rhythms, common comorbidity features in many psychiatric patients, and circadian abnormalities (including mutations in core clock genes), are associated with mood disorders such as depression and BD.^22^ However, the real impact of wakefulness and sleep on synaptic plasticity and progressive neurodegeneration awaits further confirmation.^23^

Moreover, based on the above specificities, *Dm* SH could also be suggested to model mania in terms of face, construct and predictive validity criteria.^24^ For that purpose, we have better characterized the SH mutants' model and studied the effects of Mem in *Dm* Canton‐S (WT) and SH as well as in comparison with Li^+^ in several behavioral features. Moreover, we have studied potential mechanisms encompassing both altered glutamate receptors subunits expression and the possible defects of *Shaker* channels.

## 2 MATERIALS AND METHODS

### 2.1 Animals

Adult male WT and SH mutants (Bloomington, Indiana), reared on standard cornmeal‐yeast medium, in controlled environmental conditions (25°C, 60% RH, light/dark = 12/12 h), were used. The SH phenotypes selection was conducted by using an ether‐induced leg shaking, caused by the gene mutation.

### 2.2 Drugs

Memantine HCl (Tocris) and Lithium Chloride (Sigma‐Aldrich) were dissolved in distilled water and added to the diet.

### 2.3 Survival curves

Cohorts (*n* = 80) of SH and WT were placed into vials. After their emergence from pupae, they were reared on a standard medium supplemented with Mem at different concentrations, or standard diet for control groups. Their survival rate was monitored daily. Data were collected from eclosion to death.

### 2.4 Sleep and motor activity

The effects of Mem in SH and WT flies were monitored on day 14 and 21 of treatment, and 24 h of motor activity and sleep were recorded by the Drosophila Activity Monitors System (DAMS; Trikinetics). Flies were given 24 h to adapt to the experimental conditions prior to data collection. Ten ‐ fifteen flies were gathered and placed in each monitor according to genotype (SH and WT) and experimental group (treated and controls). Locomotor activity was defined as the point in which a fly crossed and interrupted the infrared light beam in a single tube. Movement was recorded in periods of 1‐min cycles. PySolo software^25^ and a dedicated customized software package were used to elaborate data in sleep analysis. Sleep was defined as any period of uninterrupted behavioral immobility (0 counts/min), lasting >5 min.^25^

### 2.5 Nuclear magnetic resonance spectroscopy (NMR)

After 7 days of Mem‐treatment, flies were anesthetized on ice before brain dissection. Samples of 100 brains were homogenized in Deuterium Oxide and centrifuged, and 80 μL of the supernatant from each sample were analyzed on a Bruker 400 MHz spectrometer.

### 2.6 Brain and body Li^+^ concentrations

Fifty flies of both strains, untreated, and treated with 10 mM Lithium Chloride in the diet for 7 days, were used. Their heads were dissected, and their brain removed and isolated. Both bodies and brains were separately homogenized in Calf Serum, centrifuged, and the supernatant was used for the Li^+^ Assay Kit quantification (ab235613 Lithium Assay KiT; Biovision, Abcam). The ratio of the optical measurement was used to calculate Li^+^ concentration, and directly compared to its standard curve.

### 2.7 Glutamate content

Sixty flies of both strains were used. Flies' brains were isolated, homogenized, and centrifuged in an assay buffer. The supernatant was used for the Glutamate assay quantification.

Glutamate content was estimated using Cell Biolabs' Glutamate Assay Kit (STA‐674) based on a quantitative fluorometric assay, and therefore, determined by comparing the sample relative fluorescence unit (RFU) with the standard curve RFU (expressed as percentage of SH increase respect to WT).

### 2.8 Western blotting

Brains (*n* = 30) were isolated and lysed in RIPA buffer (Sigma‐Aldrich), and 10 μg of proteins were loaded onto a 4–15% SDS‐polyacrylamide gel and transferred to a PVDF membrane (Amersham Hybond P; GE Healthcare). After 30 min in 5% skimmed dry milk in TBST, membranes were incubated with the primary antibody (goat anti‐NMDAζ1, 1:500; Santa Cruz Biotechnology, sc1467 or goat anti‐actin, 1:100; Santa Cruz Biotechnology sc1616), overnight at 4°C, followed by HRP‐conjugated donkey anti‐goat IgG (1:2500; Santa Cruz Biotechnology, sc‐2020) for 1 h. Protein bands were detected with Clarity Western chemiluminescent substrate (Bio‐Rad), and signals were visualized by Image QuantTM LAS 4000. Quantitative assessment was performed by Image Studio Lite software in a densitometric analysis fashion.

### 2.9 Brain volume estimation

SH and WT naive flies (*n* = 10) were anesthetized on ice and then immersed overnight in fixative (4% paraformaldehyde). Then, the flies were embedded in PBS‐35% gelatin and their heads were sliced into 40 μm thick sections with a vibratome (Leica), mounted on slides, and processed for Nissl staining. A dedicated software (Stereologer; System Planning and Analysis, Inc.), linked to a motorized stage on the BX‐60 Olympus light microscope, was used to analyze the total brain volume using the Cavalieri method.^26^ The coefficient of error (CE) for each estimation and animal ranged from 0.05 to 0.1.

### 2.10 Body size analysis

Ten naive flies of each strain, randomly chosen, were mounted‐on slides. Images of the entire flies were captured using a Moticam 2300 (3.0 MPixel, USB 2.0) color digital camera, coupled with a stereomicroscope (Zeiss, Stemi 2000‐C), and analyzed with Motic Images Plus 2.0 ML software. The body length, from the head to the end tip of the scutellum, was measured.

### 2.11 Statistical analysis

Data are presented as means ± SEM. Two group comparisons have been analyzed by means of *t* test, while multiple comparisons have been analyzed by factorial two‐way ANOVA with the Strain and Treatment as between group factors. Before performing the above‐mentioned analyses, datasets have been inspected for normal distribution by using the Shapiro–Wilks test and for homogeneity of variances between the experimental groups with the Bartlett's test. When normal distribution of data and/or homogeneity of variances was not satisfied, statistical analyses were conducted as follows: in the case of two group comparisons, a Mann–Whitney *U* test was performed instead of a parametric *t* test; in the case of factorial analyses, datasets were at first transformed (i.e., *Y* = squared *Y*) and then inspected again for normality and homoscedasticity. This procedure allowed to obtain a normal distribution of data and homogeneous variances across the experimental groups; thus, the factorial ANOVA was applied. When two‐way ANOVA revealed statistically significant interactions, sources of significance were ascertained by pairwise post‐hoc analyses by using the HSD Tukey's test; in all the other cases pair‐wise comparisons were performed by using two‐tailed *t* tests with Bonferroni's corrected alpha values. For mortality analysis, Kaplan–Meier survival and statistical comparisons Gehan‐Breslow‐Wilcoxon test were used. Statistical analyses were all carried out with PRISM, GraphPad 8 Software, with the significance level set at *p* < 0.05.

## 3 RESULTS

### 3.1 Characterization of the SH mutant

#### 3.1.1 Motor activity and sleep

**FIGURE 1:**
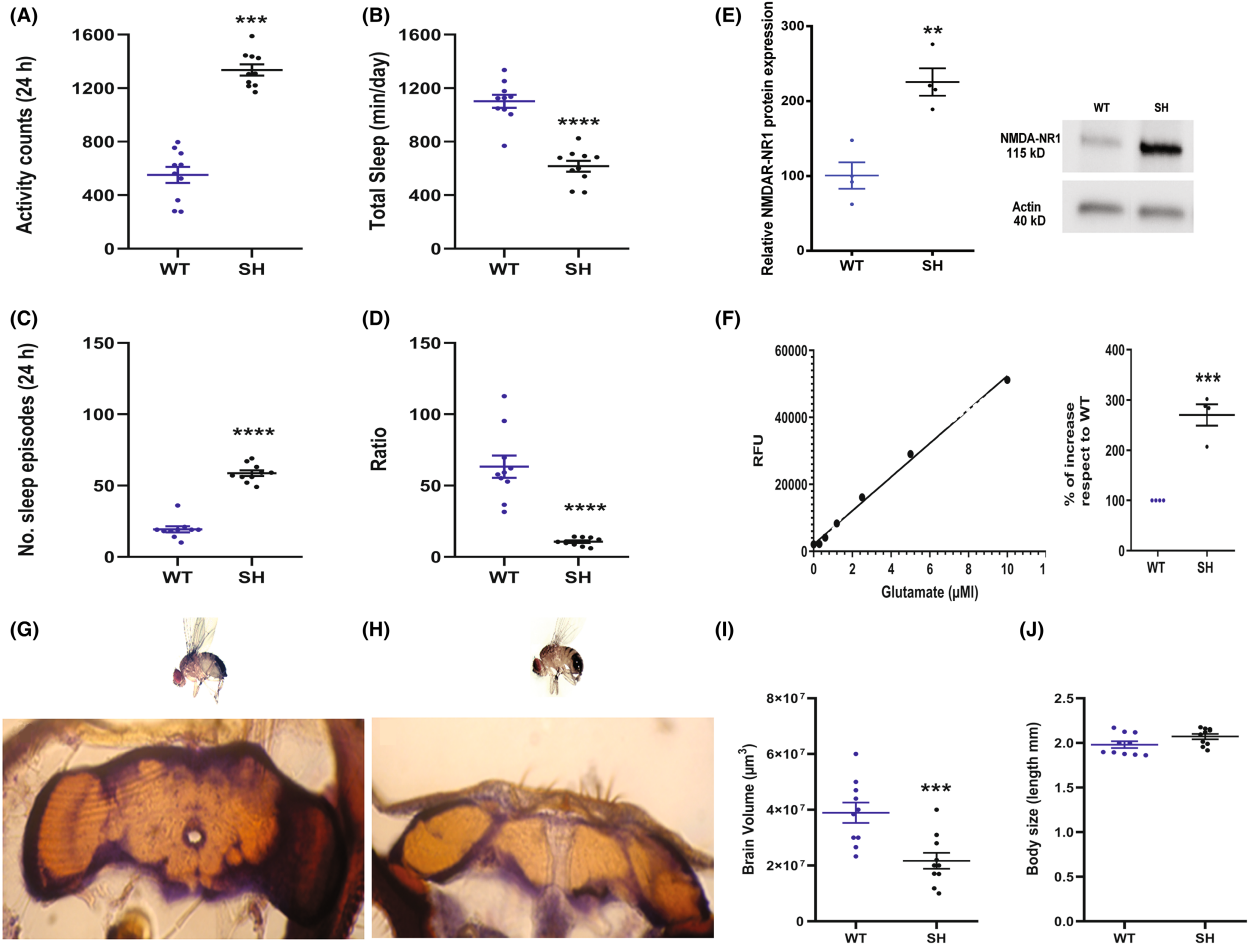
Graphs illustrate the activity (A), total sleep (B), sleep episodes (C) and ratio (D) at time zero in naïve SH and WT flies and represent the mean ± SEM (24 h recording). Ratio was calculated as total time spent asleep and number of sleep episodes. ****p < 0.0001 and ***p < 0.001 in SH versus WT (Student's *t* test). (E) NMDA‐NR1 expression in SH and WT naïve flies by Western blot analysis, semiquantitative protein expression was calculated as a ratio to β‐actin. The values are the mean ± SEM of four experiments. **p < 0.01 (Mann–Whitney test). (F) Detection of glutamate in SH and WT brains by RFU standard curve and percentage of increase with respect to WT. ***p < 0.001 (unpaired *t*‐test). Morphometric analysis of brain and body flies. (G, H) micrographs (20×) showing WT and SH brain sections stained with Nissl. Volume and body size of SH and WT brains (I, J). ***p < 0.001 (Student's *t*‐test).

SH and WT flies were tested for their basal activity and sleep pattern at time zero (*T*_0_), corresponding to 1–2 days from enclosure. According to previous studies^18^ in Figure 1A, a statistically significant (*p* < 0.001) motor activity increase was detected in mutants versus WT flies. Similarly, statistically significant differences for total sleep (Figure 1B) and sleep episode number (Figure 1C), were observed (*p* < 0.0001). With the aim to better evaluate the sleep quality throughout a 24 h period, the ratio between total time spent asleep and number of sleep episodes, was calculated (Figure 1D), resulting in robust differences between SH and WT groups (*p* < 0.0001).

#### 3.1.2 NMDAR‐NR1 expression

In Figure 1E at *T*_0_, the comparison of densitometric data on Western Blots from WT and SH brains showed a relevant increase (*p* < 0.01) of NMDAR‐NR1 subunit protein expression in SH compared to WT. Similar findings were obtained in another mutant, the Hk1, carrying an alteration in the potassium SH channel beta subunit,^27^ by comparison with those of WT for NR1 expression (Figure S2).

#### 3.1.3 Glutamate brain content

In order to prove that brain glutamate can contribute to the different behavior in SH, we proceeded in analyzing WT and SH brains for glutamate detection at *T*_0_. In SH brains we found a statistically higher presence of glutamate, expressed as a percentage of increase, over the WT strain (Figure 1F, *p* < 0.001).

#### 3.1.4 Brain and body morphometric analysis

A morphometric analysis of SH versus WT flies at *T*_0_ was carried out to understand whether the observed changes in NMDAR‐NR1 subunit protein expression and glutamate levels in mutants, can correlate with an alteration in brain size. Using the Cavalieri's method, a smaller brain size, in terms of the average brain volumes, was estimated (*p* < 0.001) in mutant SH compared to the wild type flies (Figure 1G–I). In contrast, the whole‐body size did not differ between strains (Figure 1J).

### 3.2 Drug treatment

#### 3.2.1 Effect of Mem‐treatment on SH and WT lifespan

**FIGURE 2:**
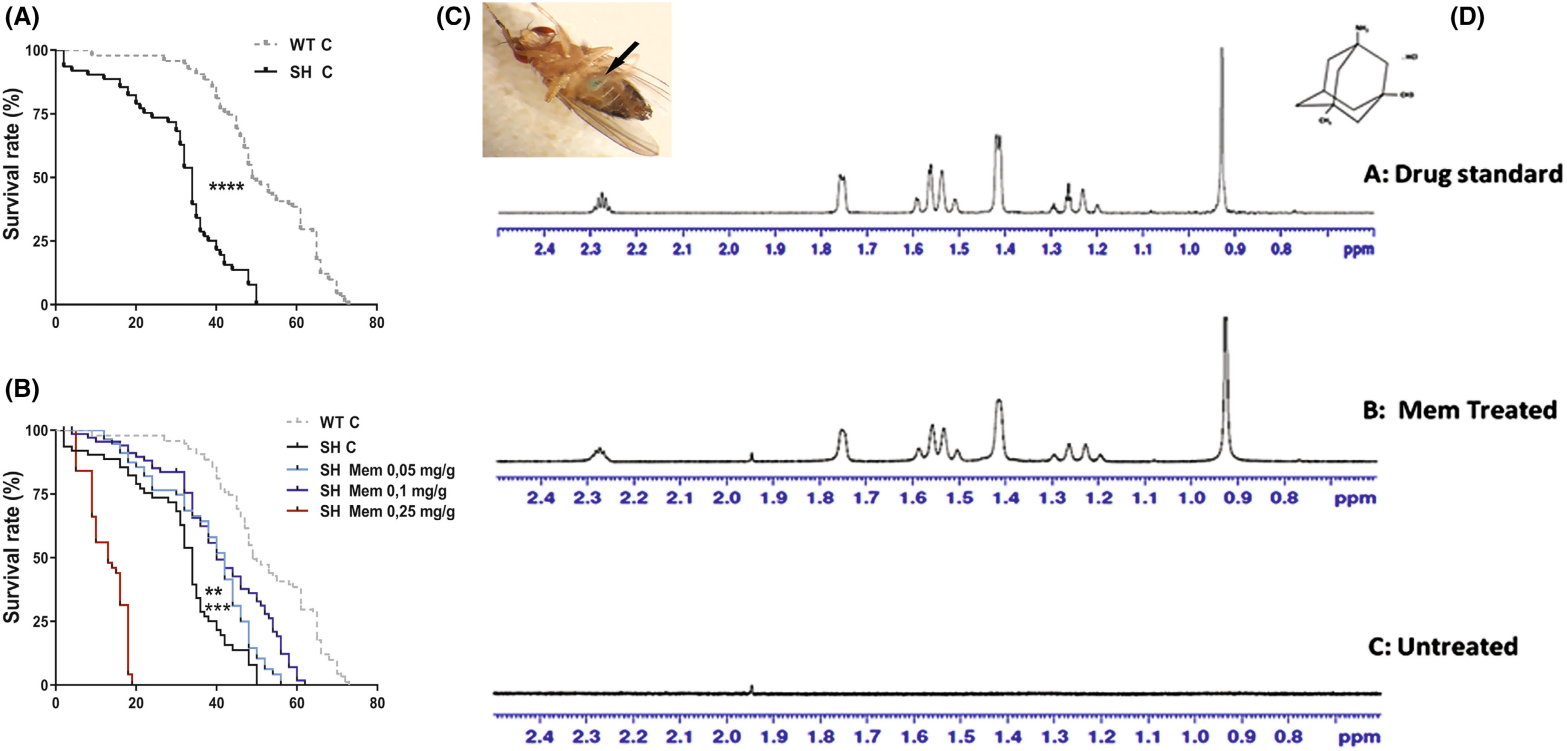
Lifespan in untreated WT and SH flies (A) and upon Mem administration at three different concentrations (B). Survival curves data are expressed as mean ± SEM. ****p < 0.0001 between SH and WT flies and between SH treated with Mem 0.1 mg/g‐diet and SH controls, ***p < 0.01 between SH treated with Mem 0.1 mg/g‐diet and SH controls **p < 0.01 between SH treated with Mem 0.05 mg/g‐diet and SH controls (Kaplan–Meier survival curves; Gehan‐Breslow‐Wilcoxon test). Blue abdomen (arrow) of Dm reared on Mem medium/blue food dye (C). (D) Overlay of 1‐H‐NMR spectra of Mem hydrochloride (0.1 mg/g‐diet) from drug standard (A) Mem‐treated (B) and untreated SH brains (C).

SH mutants displayed a shorter life span compared to WT (*p* < 0.0001, Figure 2A).

Mutant flies at 34 days after enclosure displayed a dramatic decay in their survival rate (about 50%), while about 6% were living longer, until 50 days.

For assessing Mem's ability to affect lifespan, both strains were supplied with Mem at various concentrations (0.05, 0.1, and 0.25 mg/g‐diet) and compared with non‐treated flies (Figure 2B).

No statistically significant differences between Mem‐treated and untreated mutants were noticed at the lower dose of 0.025 mg/g‐diet (data not shown).

Instead, a statistically significant difference (*p* < 0.01) between treated (0.05 mg/g‐diet) and control mutants was found, (56 days survival vs. control). Such difference was enhanced at the higher dose of 0.1 mg/g‐diet, resulting in a more statistically significant (*p* < 0.0001) survival rate (approximately 62 days vs. non‐treated ones).

Nevertheless, Mem treatment failed to affect the lifespan of WT (Figure S1). Conversely, at the highest concentration (0.25 mg/g diet), Mem was proved to be toxic in both strains (Figure 2B; Figure S1).

The evidence that all behavioral effects result from Mem treatment is shown in Figure 2C, where the blue abdomen was due to ingestion of a color‐marked Mem‐enriched medium.

The NMR analysis detected the presence of Mem in treated SH brain flies, but not in untreated ones (Figure 2D).

#### 3.2.2 Effect of Mem‐treatment on motor activity and sleep

In agreement with the results obtained on life span, experiments on motor activity and sleep parameters were performed at the effective Mem concentrations of 0.05 and 0.1 mg/g‐diet in both strains.

**FIGURE 3:**
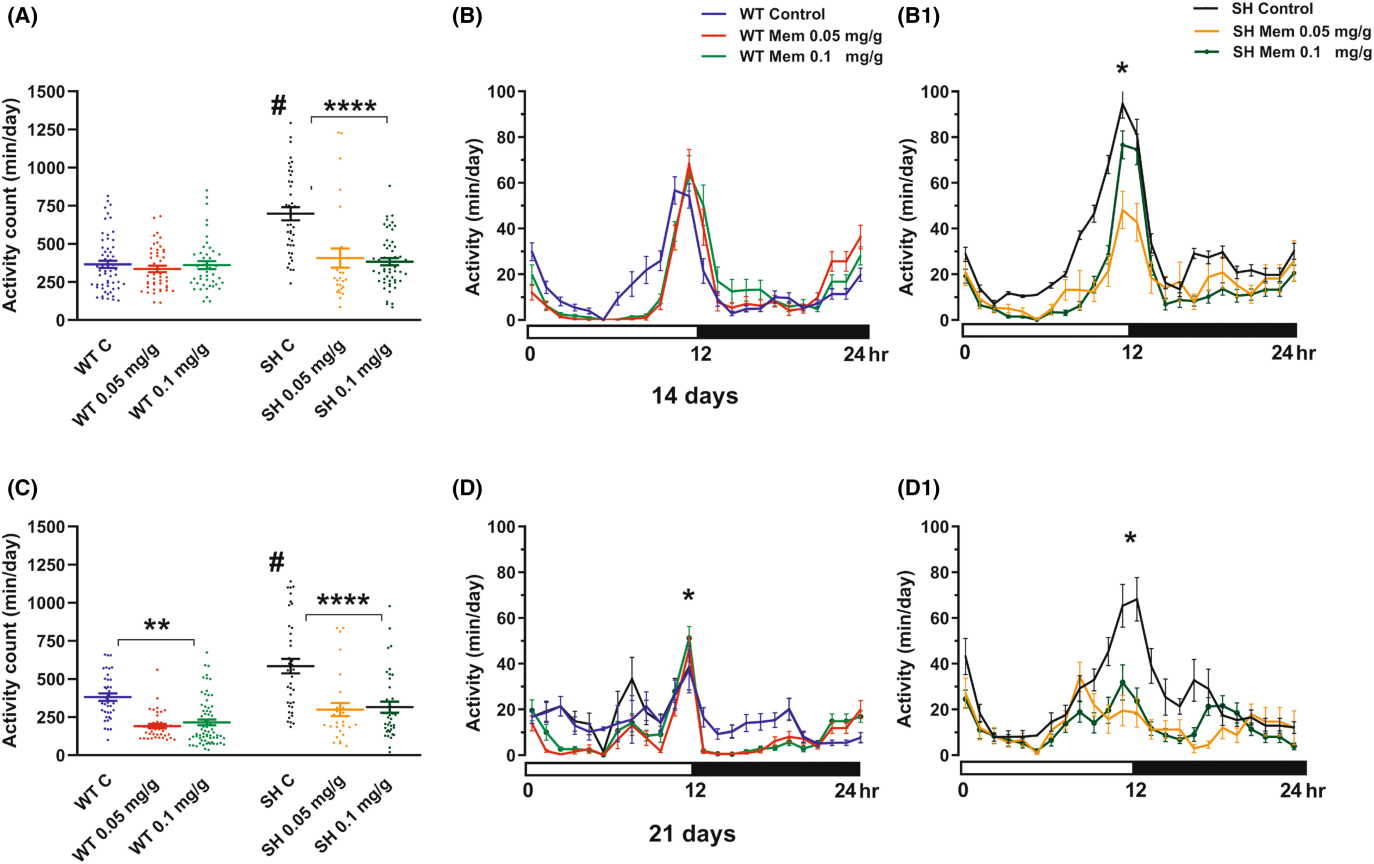
Effect of Mem treatment on activity. Graphs represent values expressed as mean ± SEM in 24 h activity recording after 14 days (A) and 21 days (C) of treatment. Graphs B‐B1 and D‐D1 illustrate the 24 h time–course activity in WT and SH flies after 14 and 21 days of Mem treatment, respectively. ^#^ *p* < 0.001 in SH control versus WT control group and ****p < 0.0001 between SH treated versus SH control group; **p < 0.01 in WT Mem treated versus WT control and *p < 0.05 in SH treated versus SH control (Tukey's multiple comparisons).

Following 14 days of Mem administration, a significant decrease of motor activity was evident for strain (*p* < 0.0001), and treatment (*p* < 0.0001) factors, as well as for strain × treatment interaction (*p* < 0.0001) (Figure 3A, Table 1). SH flies were subjected to a significant reduction of Mem induced activity at 0.05 and 0.1 mg/g‐diet. On the contrary, no statistically significant difference was measured in the motor activity of Mem‐treated WT when compared to untreated ones (*p* = 0.9663) (Figure 3A).

**TABLE 1:** Effect of Mem treatment on activity and sleep in SH and WT.

Parameter	A (2 weeks)				B (3 weeks)			
	*F* values			DF, *n*	*F* values			DF, *n*
	Strain	Treatment	Interaction		Strain	Treatment	Interaction	
Activity (24 h)	17.93****	14.54****	12.48***	1,2,2, 263	19.27****	27.15****	0.118	1,2,2, 247
Total sleep (24 h)	46.25****	65.70****	30.22****	1,2,2, 263	49.58****	56.48****	2.643	1,2,2, 239
N. sleep episode (24 h)	2.036	8.003***	2.296	1,2,2, 257	9.399**	6.211**	1.037	1,2,2, 239

*Note*: *F* values and significance levels from two‐way ANOVAs for measures performed on data reported in Figures 3A, 4A,B (A) and 3C, 4D,E (B).

****p < 0.0001; ***p < 0.001; **p < 0.01; *p < 0.05.

Motor activity was also downregulated in SH on day 21 of Mem treatment (Figure 3C, Table 1), with a statistically significant strain (*p* < 0.0001) and treatment‐dependent effect (*p* < 0.0001). No strain × treatment interaction effect was noticed, but a lower‐order one‐way ANOVA revealed a significant effect of 0.05 and 0.1 Mem mg/g‐diet in the SH mutants. Similarly, a treatment effect induced by Mem at both doses was also present in WT flies (*p* < 0.01) when compared to WT control.

The analysis of time‐course activity measured for 24 h on day 14 and 21 is illustrated in Figure 3B‐B1 and D‐D1, respectively. Interestingly, there was a common feature during the monitored period represented by a peak in motor activity from the 10 to 15‐h interval in both strains, with specific significant values always expressed at 12 h between SH treated and SH control (*p* < 0.05).

**FIGURE 4:**
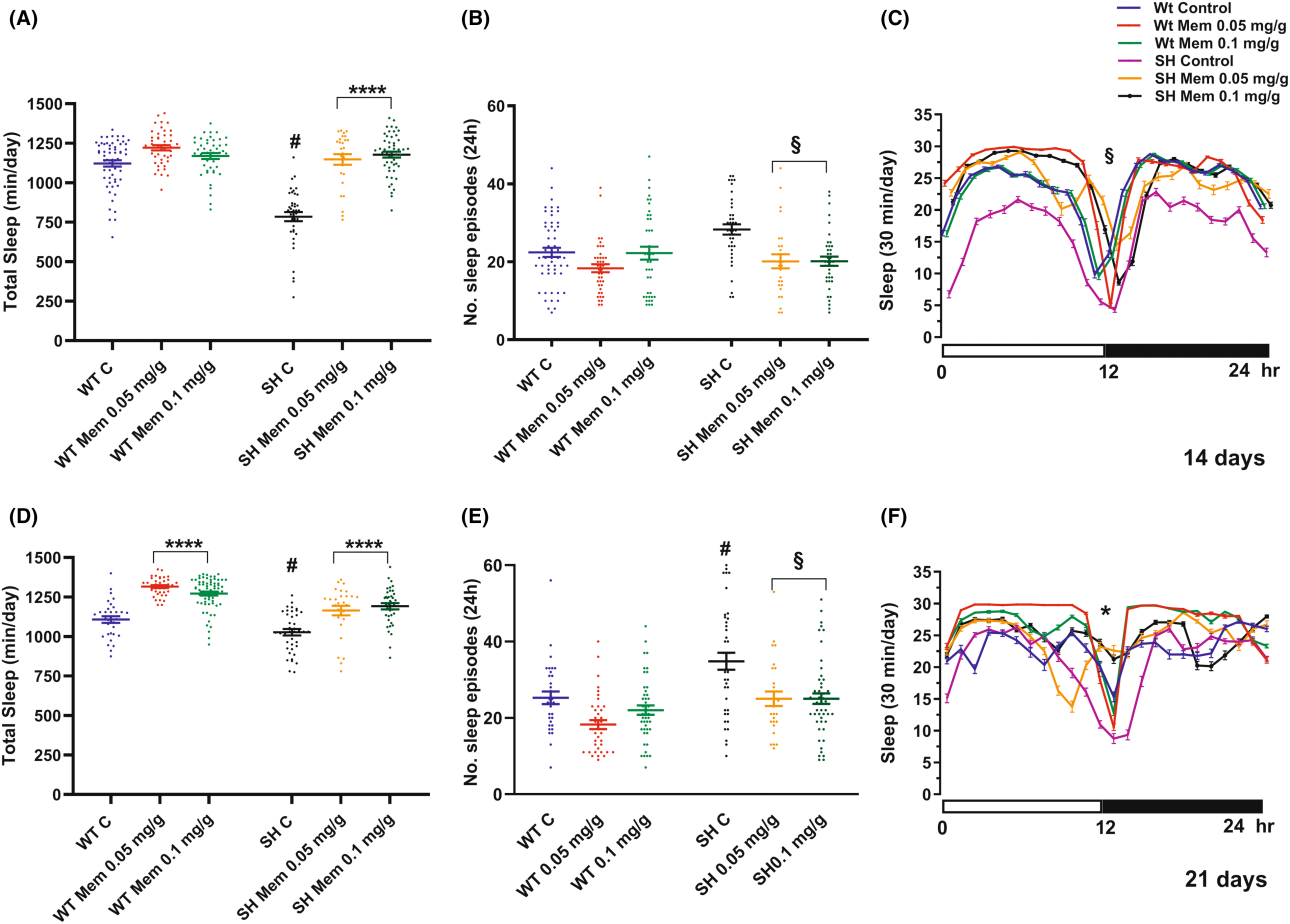
Effect of Mem treatment on total sleep and No. of sleep episodes. Graph values for total sleep (A–D) and No. of sleep episodes (B–E) are expressed as mean ± SEM in 24 h recording at 14 and 21 days of Mem‐treatment. Graphs C (14 days) and F (21 days) illustrate the daily time course (intervals of 30‐min) of the amount of sleep in treated and untreated WT and SH. ^#^ *p* < 0.001 in SH control versus WT control group; ****p < 0.0001 between SH and WT treated versus respective controls; ^§^ *p* < 0.05 SH treated versus control, *p < 0.05 SH and WT treated versus respective controls (Tukey's multiple comparisons test).

Concerning the 24‐h total sleep period, Figure 4A and Table 1 depicts significant effects, from strain (*p* < 0.0001), treatment (*p* < 0.0001), and strain × treatment interaction (*p* < 0.0001). Mem (upon 14 days of intake, both 0.05 and 0.1 mg/g‐diet), was able to increase the total sleep in SH flies compared to WT, whereas after 21 days of treatment ANOVA disclosed significant strain (*p* < 0.0001) and treatment effects (*p* < 0.0001), but not strain × treatment interaction (Figure 4D, Table 1). Mem elicited a significant increase of the total sleep time, both in WT (*p* < 0.0001) and in SH (*p* < 0.0001), with no statistically significant differences between the groups. A similar significant effect (*p* < 0.05) was evident when monitoring the overall time course for the amount of sleep (Figure 4C–F).

Regarding sleep episodes, after 14 days of Mem (Figure 4B) both no strain and no strain × treatment‐interaction was observed (Table 1). The one‐way ANOVA highlighted the specific effect of Mem when administered to SH (*p* < 0.05), whereas the drug had no statistic value in WT (*p* = 0.1909). A significant effect of strain (*p* < 0.01) and treatment (*p* < 0.01) was evident also at 21 days, while no strain × treatment interaction was detected (Table 1). More specifically, one‐way ANOVA indicated a significant effect of Mem treatment in the SH mutants (Figure 4E) (*p* < 0.05), but not in WT (*p* < 0.1595).

#### 3.2.3 Effect of Li^+^‐treatment on motor activity and sleep

To assess the presence of possible behavioral differences or analogies in comparison with Mem, adult flies from both strains were treated for a period of 14 days with Li^+^ 10 mM.^28^

**FIGURE 5:**
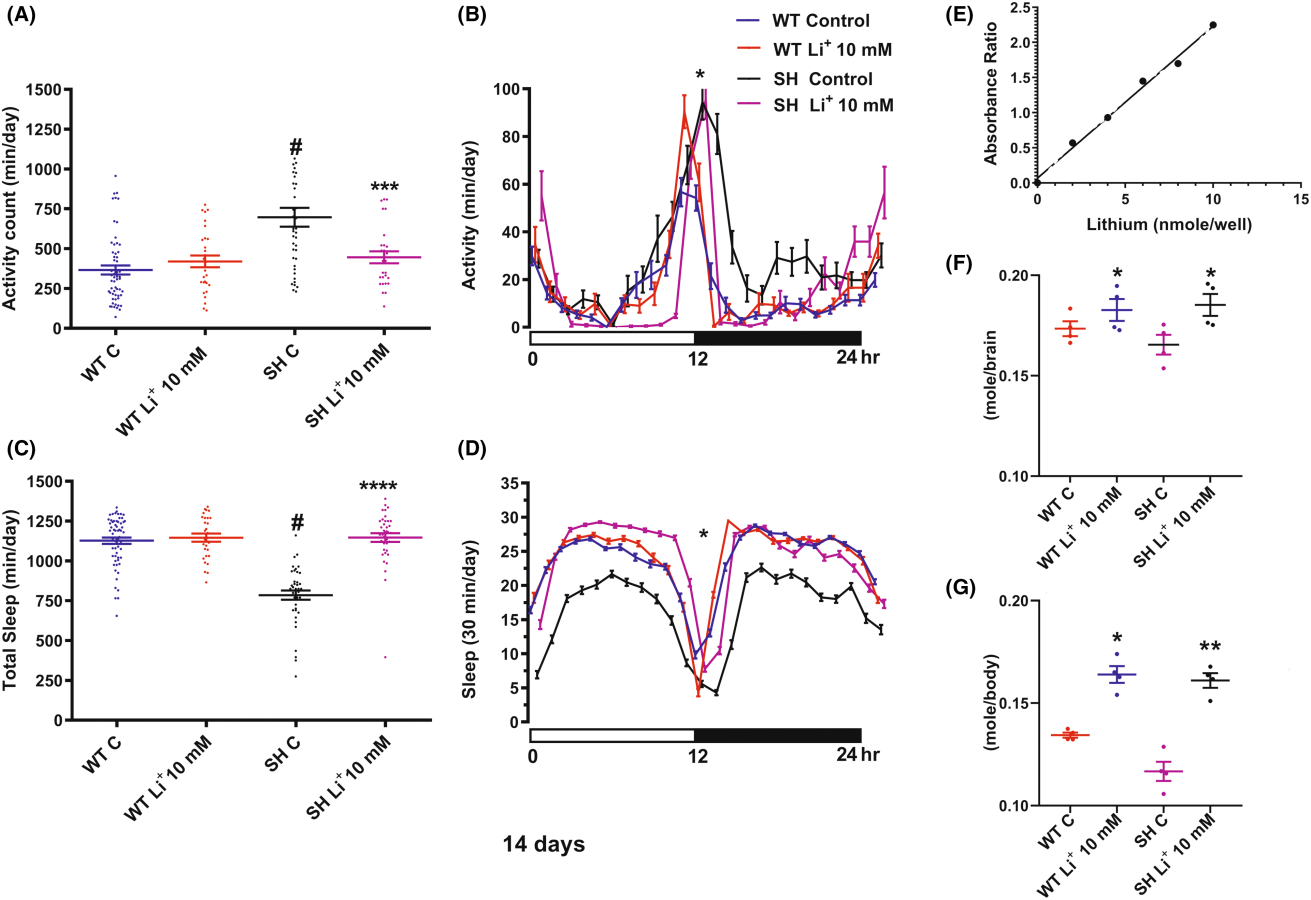
Graphs (A) and (C) illustrate the activity recording and the total sleep expressed as mean ± SEM (24 h) after 14 days of Li^+^‐treatment; (B) and (D) the daily time course in the amount of activity and sleep (30‐min interval) in 14 days Li^+^‐treated and untreated WT and SH. ****p < 0.0001 versus SH control; ***p < 0.001 versus SH control, ^#^ *p* < 0.001 versus WT group; *p < 0.05 between SH treated and respective control (Tukey's multiple comparisons test). Graph (E) represents the standard curve (ratio of the optical measurement), (F, G) depict Li^+^ concentration in brain and body respectively after Li^+^ (10 mMol) 14 days treatment. **p < 0.01 and *p < 0.05 WT and SH treated versus respective control (Tukey's multiple comparisons test).

Similar to Mem, the Li^+^‐treatment significantly reduced motor activity in mutants SH (Figure 5A, Table 2) (*p* < 0.001), but no effect was found on WT.

**TABLE 2:** Effect of LiCl on activity and total sleep in SH and WT.

Parameter	*F* values			DF, *n*
	Strain	Treatment	Interaction	
Activity (24 h)	16.31****	5.001*	11.90***	1,1,1, 152
Total sleep (24 h)	52.50****	65.41****	52.95****	1,1,1, 164

*Note*: *F* values and significance levels from two‐way ANOVAs for measures performed on data reported in Figure 5A,C.

****p < 0.0001; ***p = 0.001; *p < 0.05.

The ANOVA test for the 24 h total sleep (Figure 5C) on Li^+^ treatment showed statistically significant effects on all factors and their combination (*p* < 0.0001). Total sleep increased in Li^+^‐treated SH flies, but no significant effect was detected in WT (*p* = 0.574 Table 2). As shown in Figure 5B,D, the time course of the activity and the amount of sleep measured for 24 h, revealed a significant recovery (*p* < 0.05) of the SH treated group, with respect to untreated SH and WT.

In order to prove that Li^+^ induces the described behavioral effects by reaching flies' brains, we proceeded in analyzing WT and SH brains for Li^+^ detection and measurement. The analysis showed that drug concentration in treated brains SH and WT, was statistically higher compared to control groups (*p* < 0.05) (Figure 5F). Furthermore, the Li^+^ bodies concentration was significantly increased in Li^+^ treated SH (*p* < 0.01) and WT (*p* < 0.05) versus controls (Figure 5G).

#### 3.2.4 Effect of Mem on NMDAR‐NR1 expression

Western blot analysis of whole brains of Mem‐treated and untreated SH and WT was carried out to check the effect of Mem on the NMDAR‐NR1 protein expression.

**FIGURE 6:**
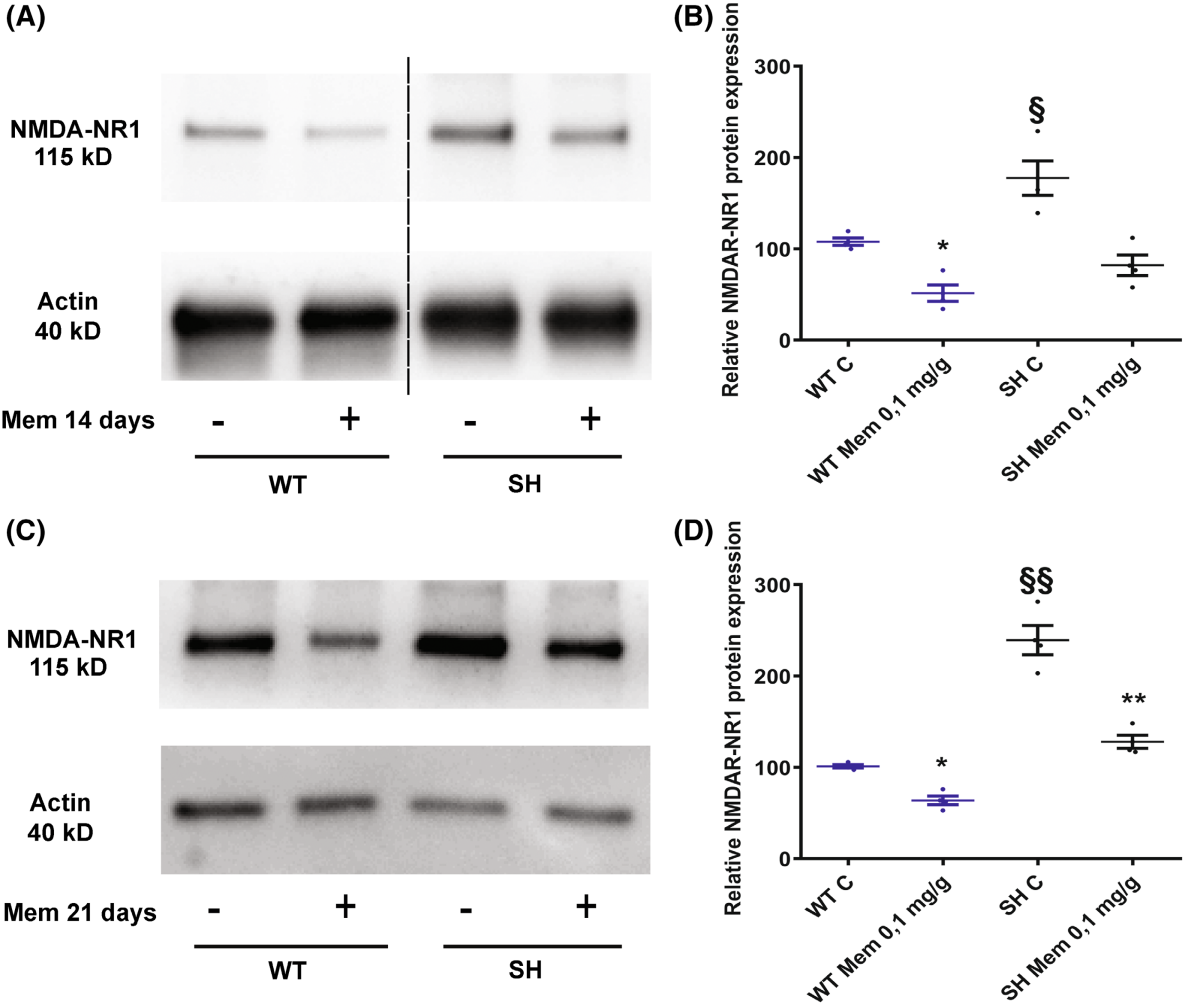
Western blotting of NMDA‐NR1 in Mem‐treated and untreated SH and WT brain flies. Representative Western blots from WT and SH flies after 14 (A) and 21 (C) days of 0.1 mg/g‐diet Mem treatment and controls. The dashed line in (A) indicates that unrelated lanes have been removed between samples. The graphs represent the expression of NMDA‐NR1 at 14 (B) and 21 (D) days of treatment and control group. The values are expressed as mean ± SEM of four experiments. *p < 0.05, **p < 0.01 mem‐treated versus controls flies. ^§^ *p* < 0.05, ^§§^ *p* < 0.01 SH versus WT both untreated (Mann–Whitney‐test).

Two‐way ANOVA on NMDAR‐NR1 receptor subunit protein expression data from flies, fed for 14 (Figure 6A,B) and 21 days (Figure 6C,D) with Mem (0.1 mg/g‐diet), revealed significant effects of strain (*p* < 0.0345 and *p* < 0.01) and treatment (*p* < 0.024 and *p* < 0.002), but not a significant strain × treatment interaction (*p* < 0.2558 and *p* = 0.0618) respectively.

A lower‐order analysis, performed by using one‐way ANOVA, indicated a significant effect on both SH (*p* < 0.01 and *p* < 0.01) and WT (*p* < 0.05 and *p* < 0.05) treatment at 14 and 21 days respectively, determining a decrease in NMDAR‐NR1 protein expression.

Original uncropped blots of replicates used for quantitative Western blot were provided in Figure S3.

## 4 DISCUSSION

Despite a shorter lifespan, behavioral disturbances of SH mutant flies encompassing reduced sleep time and motor hyperactivity, well‐replicated the same traits in most mood disorders^18^, ^19^, ^20^, ^21^, ^22^ and might be considered endophenotypes for the manic phase of BD. Therefore, in the present paper modeling genetically engineered Dm was exploited to examine Mem's benefits on behavioral activity and sleep patterns studying the neurobiology of mood disorders. Besides the overall impact of Mem on reducing the altered behavioral performances in SH mutants, the most interesting result was explicated by the extension of Mem mediated lifespan, and in association with an increase of brain glutamate (the main excitotoxic neurotransmitter), resulting in a concomitant reduction in the overexpression of the NMDAR‐NR1 subunit. Furthermore, it was observed for the first time that SH mutants brain volume is smaller when compared to WT.

In particular, there was a statistically significant elongation of the survival rate in the SH mutants, already evident upon 0.05 mg Mem, but culminating with a double dose, not observed in WT flies. In this regard, similar results with Mem have been obtained in spinocerebellar ataxia type 1 (SCA1) knock‐in (KI) mice, which mimic a progressive neurodegenerative disease caused by an extension of a CAG repeat in the Sca1 gene where Mem intake extended (about 10%) the short lifespan by possible inhibition of extra‐synaptic NMDA receptors (NMDARs).^29^

Our results clearly indicate that Mem treated flies are liable to lower their basal motor hyperactivity. This became apparent by the end of the second week, reaching a peak after 3 weeks, where the cumulative effect was also detected in the WT group. All these results agreed with the Mem mechanism of action, as an uncompetitive voltage‐dependent NMDAR antagonist which mainly inhibits a pathological state of the NMDARs when recruited extrasynaptically and activated for neuronal commitment to cell death, pointing to a determinant link with glutamate dyshomeostasis.^30^ On the other hand, it has been ascertained that Mem antagonizes the motor hyperactivity induced by ouabain administration in rats, which represents a validated model of mania or maniac phase of bipolar disorder thus far.^31^ As a matter of fact, ouabain, a potent Na/K‐ATPase inhibitor, could increase the intracellular Na^+^ and Ca^2+^ levels, glutamate presynaptic release and postsynaptic opening of NMDAR ion channels,^32^, ^33^ with consequent enhanced function of the NMDA system considering that membrane perturbations are likely present in the mutants given the Kv1.1 channel altered mechanism. Moreover, the same interpretation can be drawn assuming that Kv1.1 blockade, which is already in hippocampal slice cultures, is able to provoke an increase in glutamate release.^34^ One reasonable explanation for the exclusive neurodegenerative role of extrasynaptic NMDARs, but not synaptic NMDARs, finds a link to myotoxicity because of the documented close contact with mitochondria. Whereas postsynaptic scaffolds are typically quite distant from mitochondrial domains and, furthermore, spines usually lack mitochondria.^35^ Therefore, Mem positive contribution could result from different quantitative competition toward NMDARs differentially located in neuronal cells. Indeed, our results are coherent with the glutamate involvement in toto brain measurement of glutamate, where content accounted for about 2.5‐fold in SH when compared to WT.

Notably, in such flies, there is a high level of oxidative stress due to alterations in the metabolism of glutamate.^36^ In turn, the oxidative stress is known to activate the glutamatergic neurotransmission,^37^ which involves Mem as a putative candidate for such motor hyperactivity, considering the increased level of phosphorylated NMDA‐NR1 (GluN1S896) in sleep‐deprived mice.^38^ Another important aspect of the present study entailed both total sleep and the number of sleep episodes in SH flies, which were significantly reduced compared to the WT flies, regardless of their age. On this matter, Mem effects on different sleep patterns found in SH flies replicated those observed for motor activity with clear signs of amelioration after drug administration. In particular, Mem elicited the determinant increase of the total sleep at both day 14 and day 21 of treatment, and conversely, ameliorated the sleep quality because of the reduction in the number of sleep episodes. Such results fit well with the observations obtained in humans, although the therapeutical target was represented by Alzheimer's disease patients.^39^ The NMDAR protein expression was then examined in flies which had chronically undergone a Mem added diet. Surprisingly, the western blotting experiments showed overexpression of NMDAR1 (NR1) protein in untreated SH mutant flies compared to WT ones. In providing the simplest interpretation related to the used technique, the increased expression of the NMDA‐NR1 subunit could be attributed to the overall detection, both at synaptic and extra‐synaptic level, coming from the total protein content in the homogenate tissues.

On the other hand, this unexpected result might arise from the structural features of NMDARs, and the complexity in controlling their activity and trafficking.^2^, ^40^ As a matter of fact, NMDARs, the primary subtype of glutamate‐gated ion channels at the excitatory synapses mediating Na^+^ and Ca^++^ flow into and K^+^ ions out of the cell,^1^ is a hetero‐tetrameric plasma membrane channel made of two essential GluN1 and two modulatory GluN2 (A‐D) subunits,^41^, ^42^ with some exceptions, for example, GluN3 instead of GluN2 subunits.^43^ It is noteworthy that NMDARs are anchored to the plasma membrane by binding to more than 70 adhesion proteins,^1^ and that, among them (at the PSD95 domain), there is an interaction between the intracellular C‐terminal tails of NMDARs and the Shaker‐type K^+^ channels.^44^, ^45^ In Dm, the PSD‐95 family proteins is represented by a single homolog called Discs large (Dlg), expressed in both the nervous system and the epithelial tissues of the fly, and likewise PSD‐95, which is concentrated in synapses and tightly associated with both presynaptic and postsynaptic membrane.^45^ Dlg also binds to Dm Shaker K^+^ channels in vitro and clusters of these channels in heterologous cells.^23^, ^46^, ^47^ These previous studies have demonstrated that in sleep‐deprived Canton‐S males, both Bruchpilot protein expression – an essential constituent of the active zone of all synapses – and Dlg, were higher than in the control group, contributing to regulate not only glutamate release and postsynaptic structure,^48^ but also controlling the postsynaptic clustering of glutamatergic receptors.^49^ Thus, it is likely to assume that the SH mutants, through the alteration of the K^+^ channel function, could be related to a glutamatergic system alteration.

Interestingly, it has also been reported that Mem and low doses of ketamine were able to inhibit GSK3β, which regulates NMDARs trafficking and function, with strong implications in both BD and Li^+^ effects.^50^, ^51^ Indeed, a long‐lasting reduction of ionic and synaptic current in cortical pyramidal neurons by NMDAR intervention,^52^ has been observed. Thus, the decreased expression of NR1 subunit observed in the present study, after chronic treatment with Mem, could be explained by a minimally increased glutamatergic transmission, as demonstrated by He and Bausch,^53^ or it could be evoked by the serine‐phosphorylation upregulation of GSK3 that is known to inhibit its activity,^50^, ^51^, ^52^, ^53^, ^54^ either directly or through the mediation of AMPA receptors.^55^

In support of the aggravation of oxidative stress in the SH mutants, the observation that brains from such flies displayed a smaller volume compared to the WT ones, although the whole‐body measures tended to overlap. In favor of this, the lowered glutamate buffering capacity has been shown neurotoxic in Dm since it elicits oxidative stress and neuropil degeneration in the fly brain.^56^

Moreover, histological abnormalities, related to smaller cellular size of cerebral cortex pyramidal neurons, may be linked to the overall reduction of the brain architecture, leading to shrinkage of the gray matter involved in neurodegenerative processes elicited by glutamatergic insults.^57^, ^58^ As a matter of fact, previous data have highlighted to what extent the rate of brain atrophy is increased in neurodegenerative pathologies such as Alzheimer's disease, with neuronal loss spreading from medial temporal lobes to other regions of the brain detected by imaging. Such results provide further confirmation of the glutamate role as a possible cause of the volume reduction.^59^

Moreover, the particular phenotype in *SH* mutants (i.e., decreased sleep and increased activity), associated to the reduction of superoxide dismutase activity after sleep deprivation,^60^ could produce abnormal neurodevelopment,^61^ dictated by the glutamatergic perturbations strictly depending on the mutated Shaker channels. On insufficient sleep, there is a similar trend in healthy human brains that are severely affected in the morphometric parameters with enhanced deterioration of neuronal plasticity,^62^, ^63^ and even though abnormal cortical volumes have been found in bipolar patients,^64^ a general consensus has yet to emerge.

In conclusion, in terms of translational medicine and psychiatry, so far, few pharmacological agents can target the glutamatergic system and have indication for the treatment of BD. Only S‐ketamine has been recently approved (2019) in the United States by FDA and developed as a nasal spray formulation, despite its specific use against treatment‐resistant depression. For the glutamatergic system, lamotrigine and Mem seem to ameliorate the BD symptoms, although only the first has official indication.^65^ Because of that, the reported results may offer a promising start‐up for the therapeutic use of Mem owing to its uncommon ability to improve motor activity and sleep parameters based on this simple model and providing some quantitative indications for the new mechanism of action. These indications are also validated by the comparative experiments with Li^+^, widely used as an antimanic and mood‐stabilizing agent in BD.

Furthermore, this data, could validate Dm mutants as an alternative model to resolve some key issues concerning mood disorder, and at the same time, afford new mechanisms for better developing therapeutic agents. Finally, it must be pointed out, sleep disturbances are important early pathognomonic “markers” for several neuropsychiatric diseases, including BD.^66^ The opportunity to study alterations of sleep patterns in simple organisms such as the Dm, could represent a robust aid to predict and diagnose human dysfunctions at the beginning of their appearance.

## AUTHOR CONTRIBUTIONS

M. Collu, M.D. Setzu conceived and designed the study. F. Cadeddu, M. Fanti, M. Collu, M.D. Setzu, V. Sogos, M.A. Casu, I. Mocci performed the experiments. M. Collu, M.D. Setzu, A. Liscia, M.A. Casu, I. Mocci, V. Sogos analyzed and discussed the data. M. Collu, M.D. Setzu, A. Diana, M.A. Casu, I. Mocci, V. Sogos wrote the paper. P. Muroni, G. Talani revised the manuscript. All authors reviewed and approved the submitted version.

## CONFLICT OF INTEREST STATEMENT

There are no conflicts among the authors.

## Supporting information


Figure S1

Figure S2

Figure S3

Data S1

## Data Availability

Data are available to researchers on request for purposes of reproducing the results or replicating the procedure by directly contacting the corresponding author.

## References

[1] Fan X , Jin WY , Wang YT . The NMDA receptor complex: a multifunctional machine at the glutamatergic synapse. Front Cell Neurosci. 2014;8:160. 24959120 10.3389/fncel.2014.00160 PMC4051310

[2] Lau CG , Zukin RS . NMDA receptor trafficking in synaptic plasticity and neuropsychiatric disorders. Nat Rev Neurosci. 2007;8:413‐426. 17514195 10.1038/nrn2153

[3] de Bartolomeis A , Buonaguro EF , Iasevoli F , Tomasetti C . The emerging role of dopamine–glutamate interaction and of the postsynaptic density in bipolar disorder pathophysiology: implications for treatment. J Psychopharmacol. 2014;28:505‐526. 24554693 10.1177/0269881114523864

[4] APA Work Group on Psychiatric Evaluation . The American Psychiatric Association practice guidelines for the psychiatric evaluation of adults / APA. 3rd ed. Work Group on Psychiatric Evaluation, Joel J. Silverman, chair [and eleven others]. American Psychiatric Association; 2016.

[5] Sepede G , Chiacchiaretta P , Gambia F , et al. Bipolar disorder with and without a history of psychotic features: fMRI correlates of sustained attention. Prog Neuropsychopharmacol Biol Psychiatry. 2020;98:109817. 31756418 10.1016/j.pnpbp.2019.109817

[6] Fornaro M , Kardash L , Novello S , et al. Progress in bipolar disorder drug design toward the development of novel therapeutic targets: a clinician's perspective. Expert Opin Drug Discov. 2018;13:221‐228. 29357703 10.1080/17460441.2018.1428554

[7] Alda M . Lithium in the treatment of bipolar disorder: pharmacology and pharmacogenetics. Mol Psychiatry. 2015;20:661‐670. 25687772 10.1038/mp.2015.4 PMC5125816

[8] Kirshenbaum GS , Clapcote SJ , Duffy S , et al. Mania‐like behavior induced by genetic dysfunction of the neuron‐specific Na+,K+‐ATPase α3 sodium pump. Proc Natl Acad Sci USA. 2011;108(44):18144‐18149. Erratum in: Proc Natl Acad Sci USA. 2012;109(6):2174. 22025725 10.1073/pnas.1108416108 PMC3207708

[9] Hashimoto K , Sawa A , Iyo M . Increased levels of glutamate in brains from patients with mood disorders. Biol Psychiatry. 2007;62:1310‐1316. 17574216 10.1016/j.biopsych.2007.03.017

[10] Lan MJ , McLoughlin GA , Griffin JL , et al. Metabonomic analysis identifies molecular changes associated with the pathophysiology and drug treatment of bipolar disorder. Mol Psychiatry. 2009;14:269‐279. 18256615 10.1038/sj.mp.4002130

[11] Ongür D , Jensen JE , Prescot AP , et al. Abnormal glutamatergic neurotransmission and neuronal‐glial interactions in acute mania. Biol Psychiatry. 2008;64:718‐726. 18602089 10.1016/j.biopsych.2008.05.014 PMC2577764

[12] van Marum RJ . Update on the use of memantine in Alzheimer's disease. Neuropsychiatr Dis Treat. 2009;5:237‐247. 19557118 10.2147/ndt.s4048 PMC2695219

[13] Serra G , Demontis F , Serra F , et al. Memantine: new prospective in bipolar disorder treatment. World J Psychiatry. 2014;4:80‐90. 25540723 10.5498/wjp.v4.i4.80 PMC4274590

[14] Chen G , Henter ID , Manji HK . Partial rodent genetic models for bipolar disorder. Curr Top Behav Neurosci. 2011;5:89‐106. 25236551 10.1007/7854_2010_63 PMC4290018

[15] Einat H . New ways of modeling bipolar disorder. Harv Rev Psychiatry. 2014;22:348‐352. 25377608 10.1097/HRP.0000000000000059

[16] van Alphen B , van Swinderen B . Drosophila strategies to study psychiatric disorders. Brain Res Bull. 2013;92:1‐11. 21978945 10.1016/j.brainresbull.2011.09.007

[17] Maccioni R , Setzu MD , Talani G , et al. Standardized phytotherapic extracts rescue anomalous locomotion and electrophysiological responses of TDP‐43 *Drosophila melanogaster* model of ALS. Sci Rep. 2018;8:16002. 30375462 10.1038/s41598-018-34452-1 PMC6207707

[18] Cirelli C , Bushey D , Hill S , et al. Reduced sleep in Drosophila Shaker mutants. Nature. 2005;434:1087‐1092. 15858564 10.1038/nature03486

[19] Tanouye MA , Ferrus A . Action potentials in normal and Shaker mutant Drosophila. J Neurogenet. 1985;2:253‐271. 3936906 10.3109/01677068509102322

[20] D'Adamo MC , Liantonio A , Rolland JF , Pessia M , Imbrici P . Kv1.1 channelopathies: pathophysiological mechanisms and therapeutic approaches. Int J Mol Sci. 2020;21(8):2935. 32331416 10.3390/ijms21082935 PMC7215777

[21] Cirelli C . The genetic and molecular regulation of sleep: from fruit flies to humans. Nat Rev Neurosci. 2009;10:549‐560. 19617891 10.1038/nrn2683 PMC2767184

[22] McClung CA . Role for the clock gene in bipolar disorder. Cold Spring Harb Symp Quant Biol. 2007;72:637‐644. 18419323 10.1101/sqb.2007.72.031

[23] Gilestro GF , Tononi G , Cirelli C . Widespread changes in synaptic markers as a function of sleep and wakefulness in Drosophila. Science. 2009;324:109‐112. 19342593 10.1126/science.1166673 PMC2715914

[24] Belzung C , Lemoine M . Criteria of validity for animal models of psychiatric disorders: focus on anxiety disorders and depression. Biol Mood Anxiety Disord. 2011;1(1):9. 22738250 10.1186/2045-5380-1-9 PMC3384226

[25] Gilestro GF , Cirelli C . pySolo: a complete suite for sleep analysis in Drosophila. Bioinformatics. 2009;25(11):1466‐1467. 19369499 10.1093/bioinformatics/btp237 PMC2732309

[26] Mouton PR . Principles and practices of unbiased stereology. The Johns Hopkins University Press; 2002.

[27] Bushey D , Huber R , Tononi G , Cirelli C . Drosophila hyperkinetic mutants have reduced sleep and impaired memory. J Neurosci. 2007;27:5384‐5393. 17507560 10.1523/JNEUROSCI.0108-07.2007 PMC6672338

[28] Dokucu ME , Yu L , Taghert PH . Lithium‐ and valproate‐induced alterations in circadian locomotor behavior in Drosophila. Neuropsychopharmacology. 2005;30:2216‐2224. 15956996 10.1038/sj.npp.1300764

[29] Iizuka A , Nakamura K , Hirai H . Long‐term oral administration of the NMDA receptor antagonist memantine extends life span in spinocerebellar ataxia type 1 knock‐in mice. Neurosci Lett. 2015;592:37‐41. 25725171 10.1016/j.neulet.2015.02.055

[30] Alam S , Lingenfelter KS , Bender AM , Lindsley CW . Classics in chemical neuroscience: memantine. ACS Chem Nerosci. 2017;8:1823‐1829. 10.1021/acschemneuro.7b00270 28737885

[31] Gao Y , Payne RS , Schurr A , et al. Memantine reduces mania‐like symptoms in animal models. Psychiatry Res. 2011;188:366‐371. 21269711 10.1016/j.psychres.2010.12.030

[32] Li S , Stys PK . Na^+^‐K^+^‐ATPase inhibition and depolarization induce glutamate release via reverse Na^+^‐dependent transport in spinal cord white matter. Neuroscience. 2001;107:675‐683. 11720790 10.1016/s0306-4522(01)00385-2

[33] Veldhuis WB , van der Stelt M , Delmas F , et al. In vivo excitotoxicity induced by ouabain, a Na^+^/K^+^‐ATPase inhibitor. J Cereb Blood Flow Metab. 2003;23:62‐74. 12500092 10.1097/01.WCB.0000039287.37737.50

[34] Zbili M , Ramaa S , Benitez MJ , et al. Homeostatic regulation of axonal Kv1.1 channels accounts for both synaptic and intrinsic modifications in the hippocampal CA3 circuit. Proc Natl Acad Sci USA. 2021;118(47):e2110601118. 34799447 10.1073/pnas.2110601118 PMC8617510

[35] Li Z , Okamoto K , Hayashi Y , Sheng M . The importance of dendritic mitochondria in the morphogenesis and plasticity of spines and synapses. Cell. 2004;119(6):873‐887. 15607982 10.1016/j.cell.2004.11.003

[36] Gerhard DM , Wohleb ES , Duman RS . Emerging treatment mechanisms for depression: focus on glutamate and synaptic plasticity. Drug Discov Today. 2016;21:454‐464. 26854424 10.1016/j.drudis.2016.01.016 PMC4803609

[37] Calabrese F , Guidotti G , Molteni R , Racagni G , Mancini M , Riva MA . Stress‐induced changes of hippocampal NMDA receptors: modulation by duloxetine treatment. PLoS One. 2012;7:e37916. 22666412 10.1371/journal.pone.0037916 PMC3362535

[38] Szabo ST , Machado‐Vieira R , Yuan P , et al. Glutamate receptors as targets of protein kinase C in the pathophysiology and treatment of animal models of mania. Neuropharmacology. 2009;56:47‐55. 18789340 10.1016/j.neuropharm.2008.08.015 PMC2789350

[39] Ishikawa I , Shinno H , Ando N , Mori T , Nakamura Y . The effect of memantine on sleep architecture and psychiatric symptoms in patients with Alzheimer's disease. Acta Neuropsychiatr. 2016;28:157‐164. 26572055 10.1017/neu.2015.61

[40] Groc L , Bard L , Choquet D . Surface trafficking of N‐methyl‐D‐aspartate receptors: physiological and pathological perspectives. Neuroscience. 2009;158:4‐18. 18583064 10.1016/j.neuroscience.2008.05.029

[41] Cull‐Candy S , Brickley S , Farrant M . NMDA receptor subunits: diversity, development and disease. Curr Opin Neurobiol. 2001;11:327‐335. 11399431 10.1016/s0959-4388(00)00215-4

[42] Collingridge GL , Olsen RW , Peters J , Spedding M . A nomenclature for ligand‐gated ion channels. Neuropharmacology. 2009;56:2‐5. 18655795 10.1016/j.neuropharm.2008.06.063 PMC2847504

[43] Ulbrich MH , Isacoff EY . Rules of engagement for NMDA receptor subunits. Proc Natl Acad Sci USA. 2008;105:14163‐14168. 18779583 10.1073/pnas.0802075105 PMC2544595

[44] Kim E , Niethammer M , Rothschild A , Jan YN , Sheng M . Clustering of Shaker‐type K+ channels by interaction with a family of membrane‐associated guanylate kinases. Nature. 1995;378:85‐88. 7477295 10.1038/378085a0

[45] Sheng M , Wyszynski M . Ion channel targeting in neurons. Bioessays. 1997;19:847‐853. 9363678 10.1002/bies.950191004

[46] Tejedor FJ , Bokhari A , Rogero O , et al. Essential role for dlg in synaptic clustering of Shaker K1 channels In vivo. J Neurosci. 1997;17:152‐159. 8987744 10.1523/JNEUROSCI.17-01-00152.1997 PMC4658234

[47] Ruiz‐Cañada C , Koh YH , Budnik V , Tejedor FJ . DLG differentially localizes Shaker K+‐channels in the central nervous system and retina of Drosophila. J Neurochem. 2002;82:1490‐1501. 12354297 10.1046/j.1471-4159.2002.01092.x

[48] Budnik V , Koh YH , Guan B , et al. Regulation of synapse structure and function by the Drosophila tumor suppressor gene dlg. Neuron. 1996;17:627‐640. 8893021 10.1016/s0896-6273(00)80196-8 PMC4661176

[49] Chen K , Featherstone DE . Discs‐large (DLG) is clustered by presynaptic innervation and regulates postsynaptic glutamate receptor subunit composition in Drosophila. BMC Biol. 2005;3:1. 15638945 10.1186/1741-7007-3-1 PMC545058

[50] De Sarno P , Bijur GN , Zmijewska AA , Li X , Jope RS . In vivo regulation of GSK3 phorphorylation by cholinergic and NMDA receptors. Neurobiol Aging. 2006;27:413‐422. 16464655 10.1016/j.neurobiolaging.2005.03.003 PMC1618800

[51] Beurel E , Song L , Jope RS . Inhibition of glycogen synthase kinase‐3 is necessary for the rapid antidepressant effect of ketamine in mice. Mol Psychiatry. 2011;11:1068‐1070. 10.1038/mp.2011.47 PMC3200424 21502951

[52] Chen P , Gu Z , Liu W , Yan Z . Glycogen synthase kinase 3 regulates N‐methyl‐D‐aspartate receptor channel trafficking and function in cortical neurons. Mol Pharmacol. 2007;72:40‐51. 17400762 10.1124/mol.107.034942

[53] He S , Bausch SB . Synaptic plasticity in glutamatergic and GABAergic neurotransmission following chronic memantine treatment in an in vitro model of limbic epileptogenesis. Neuropharmacology. 2014;77:379‐386. 24184417 10.1016/j.neuropharm.2013.10.016 PMC3880158

[54] Luo HR , Hattori H , Hossain MA , et al. Akt as a mediator of cell death. Proc Natl Acad Sci USA. 2003;100:11712‐11717. 14504398 10.1073/pnas.1634990100 PMC208823

[55] Nishimoto T , Kihara T , Akaike A , Niidome T , Sugimoto H . AMPA reduced surface expression of NR1 through regulation of GSK3β. Neurochemistry. 2009;20:161‐165. 10.1097/WNR.0b013e3283118450 19151600

[56] Rival T , Soustelle L , Strambi C , Besson MT , Iché M , Birman S . Decreasing glutamate buffering capacity triggers oxidative stress and neuropil degeneration in the Drosophila brain. Curr Biol. 2004;14:599‐605. 15062101 10.1016/j.cub.2004.03.039

[57] Kumar A , Babu G . In vivo neuroprotective effects of peripheral kynurenine on acute neurotoxicity induced by glutamate in rat cerebral cortex. Neurochem Res. 2010;35:636‐644. 20035383 10.1007/s11064-009-0114-6

[58] Kumar A , Singh R , Babu G . Cell death mechanisms in the early stages of acute glutamate neurotoxicity. Neurosci Res. 2010;66:271‐278. 19944120 10.1016/j.neures.2009.11.009

[59] Smith AD . Imaging the progression of Alzheimer pathology through the brain. Proc Natl Acad Sci USA. 2002;99:4135‐4137. 11929987 10.1073/pnas.082107399 PMC123611

[60] Ramanathan L , Gulyani S , Nienhuis R , Siegel JM . Sleep deprivation decreases superoxide dismutase activity in rat hippocampus and brainstem. Neuroreport. 2002;13:1387‐1390. 12167758 10.1097/00001756-200208070-00007 PMC8802885

[61] Camm EJ , Tijsseling D , Richter HG , et al. Giussani DA oxidative stress in the developing brain: effects of postnatal glucocorticoid therapy and antioxidants in the rat. PLoS One. 2011;6:e21142. 21698270 10.1371/journal.pone.0021142 PMC3115992

[62] Sun J , Zhao R , Yang X , et al. Alteration of brain gray matter density after 24 h of sleep deprivation in healthy adults. Front Neurosci. 2020;14:754. 32903801 10.3389/fnins.2020.00754 PMC7438917

[63] Dai XJ , Jiang J , Zhang Z , et al. Plasticity and susceptibility of brain morphometry alterations to insufficient sleep. Front Psych. 2018;9:266. 10.3389/fpsyt.2018.00266 PMC6030367 29997530

[64] Hibar DP , Westlye LT , Doan NT , et al. Cortical abnormalities in bipolar disorder: an MRI analysis of 6503 individuals from the ENIGMA Bipolar Disorder Working Group. Mol Psychiatry. 2018;23:932‐942. 28461699 10.1038/mp.2017.73 PMC5668195

[65] Karakatsoulis GN , Fountoulakis KN . Glutamate and neuropsychiatric disorders. Current and emerging treatments. Pavlovic ZM, ed. Springer Nature; 2022: 261 p.

[66] Li JZ , Bunney BG , Meng F , et al. Circadian patterns of gene expression in the human brain and disruption in major depressive disorder. Proc Natl Acad Sci USA. 2013;110:9950‐9955. 23671070 10.1073/pnas.1305814110 PMC3683716

