# Supplementary figures and images for: Effects of memantine on mania‐like phenotypes exhibited by Drosophila Shaker mutants
Source: CNS Neurosci Ther. 2023 Mar 21;29(7):1750–61. doi: 10.1111/cns.14145 (PMC10324369; doi:10.1111/cns.14145)

WT Control  
WT Mem 0.025 mg/g  
WT Mem 0.05 mg/g  
WT Mem 0.1 mg/g  
WT Mem 0,25 mg/g

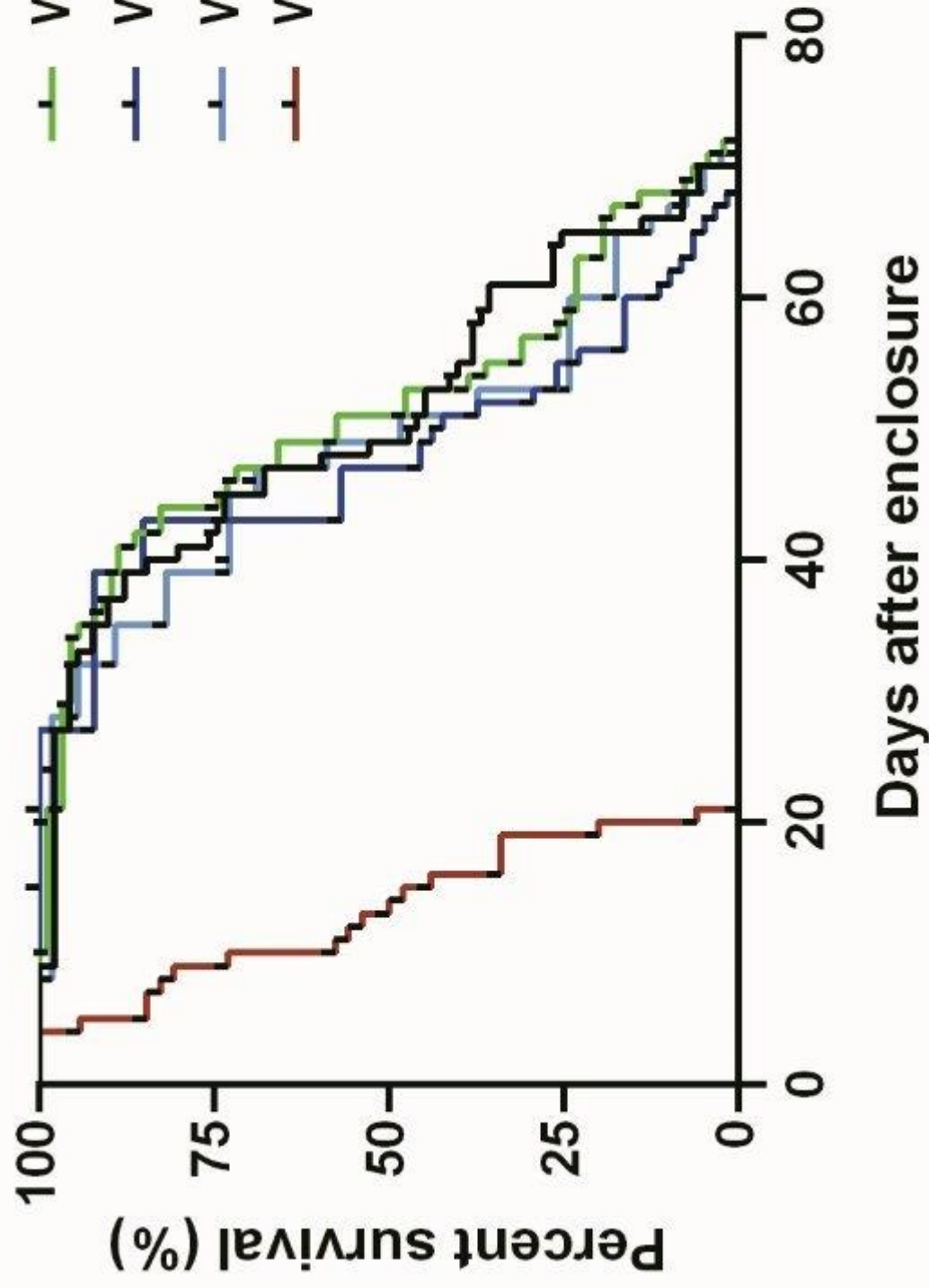

Supplement: Supplementary file 1 — Figure S1 [file CNS-29-1750-s001.pdf]

**NMDA-NR1**

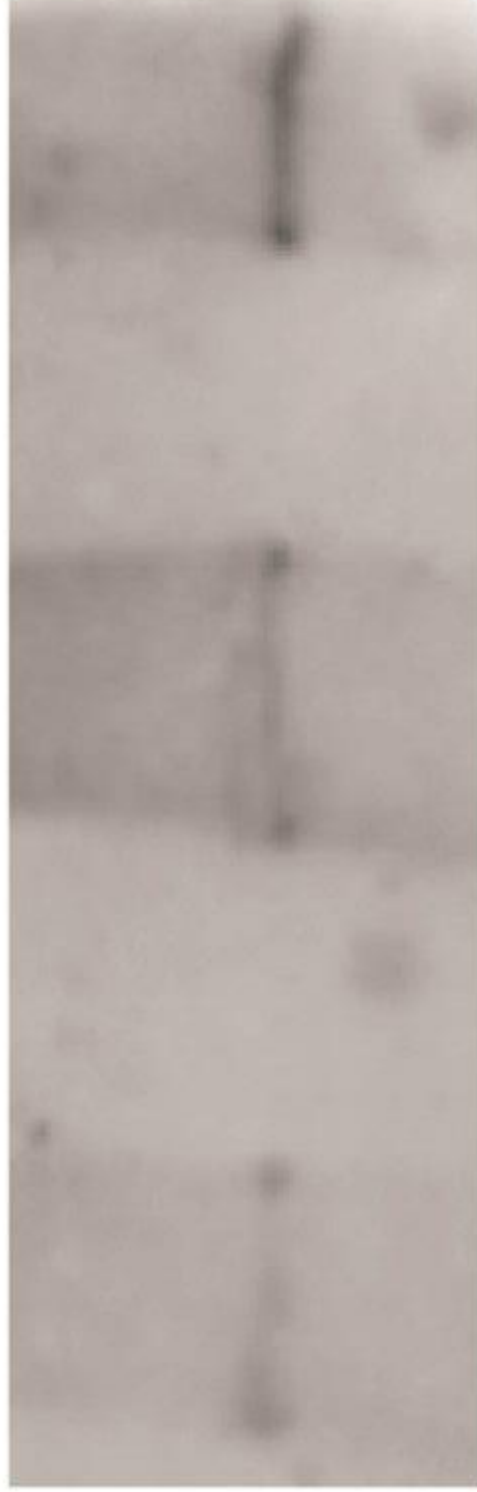

**Actin**

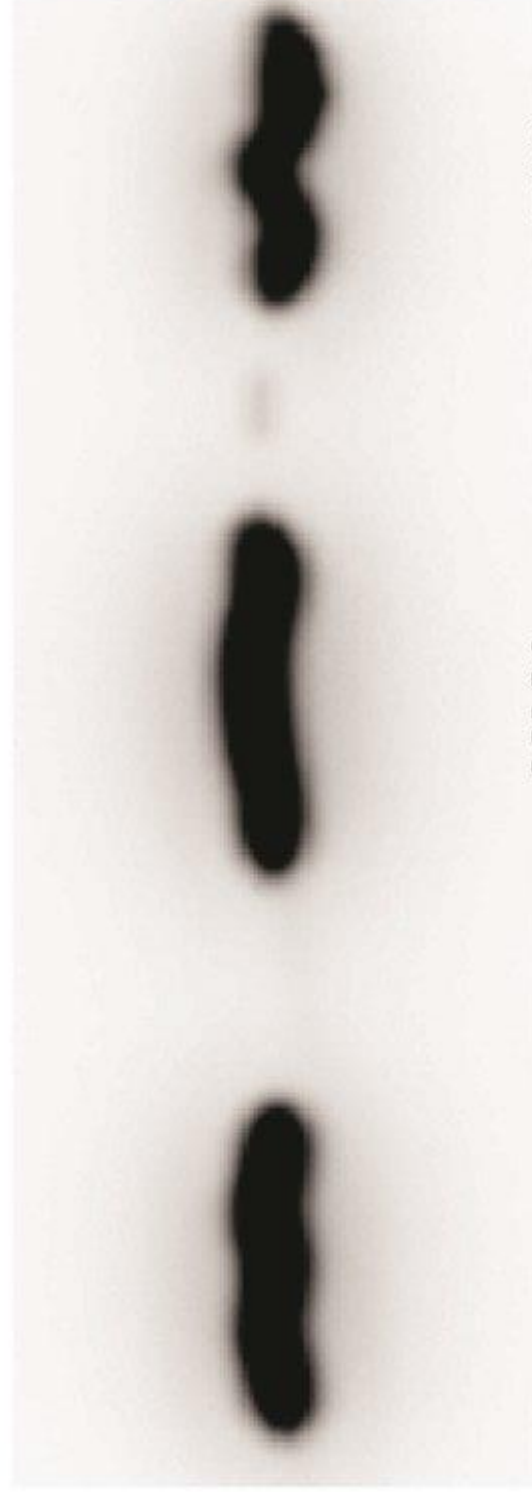

**SH**

**HK1**

**WT**

Supplement: Supplementary file 2 — Figure S2 [file CNS-29-1750-s004.pdf]

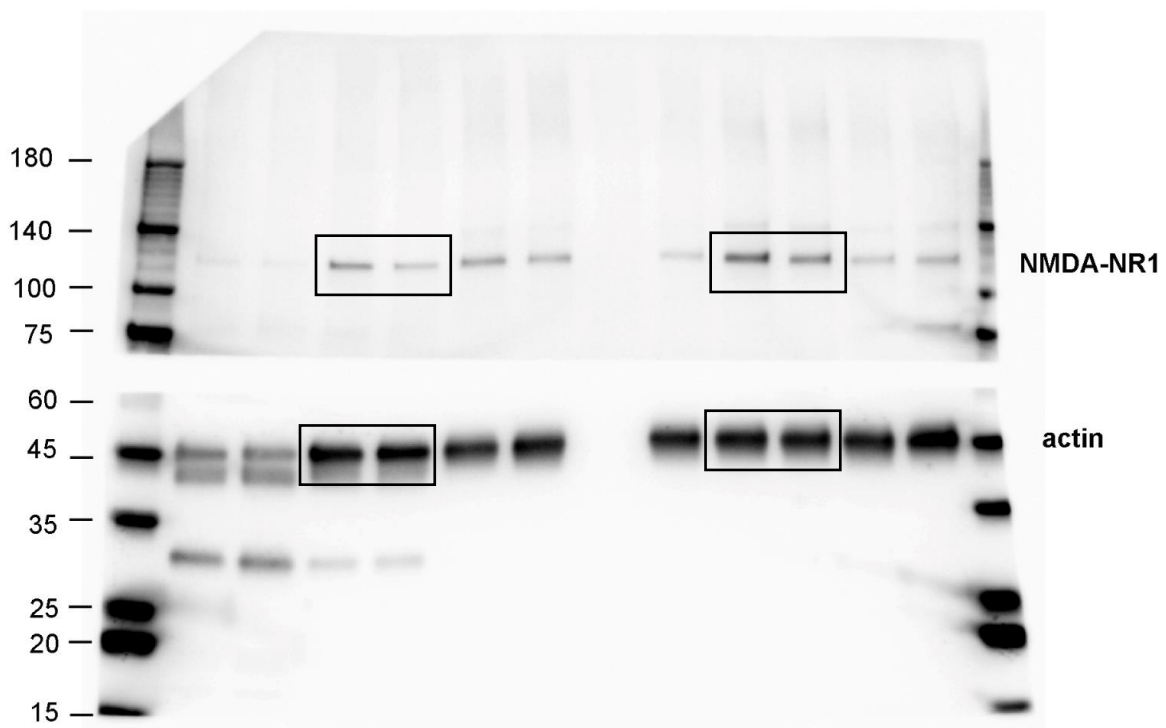

Full unedited blot for fig. 6 A

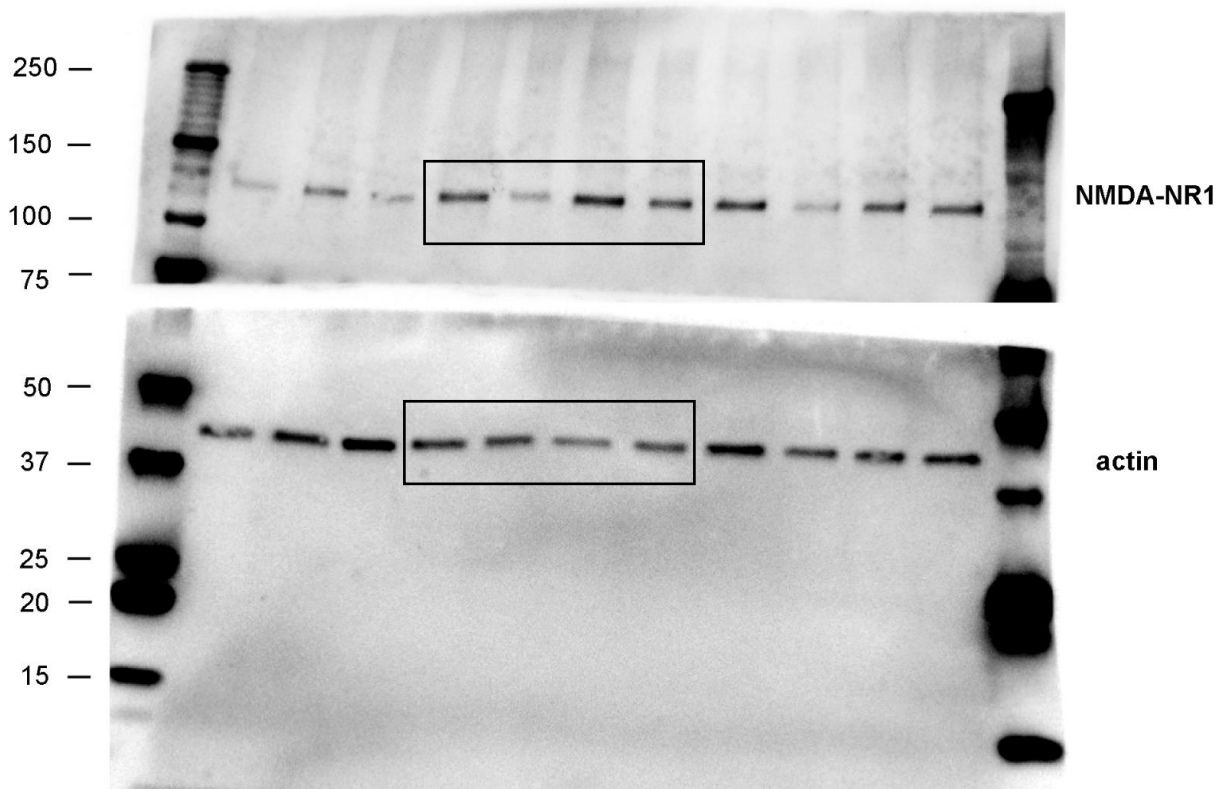

Full unedited blot for fig. 6 C

Supplement: Supplementary file 3 — Figure S3 [file CNS-29-1750-s002.pdf]
