# Supplementary material for: Effects of memantine on mania‐like phenotypes exhibited by Drosophila Shaker mutants
Source: CNS Neurosci Ther. 2023 Mar 21;29(7):1750–61. doi: 10.1111/cns.14145 (PMC10324369; doi:10.1111/cns.14145)
Supplement: Supplementary file 4 — Data S1 [file CNS-29-1750-s003.docx]

Supplementary Figure S1: Lifespan in untreated and Mem-treated WT (0.1 mg/ g-diet). Data is expressed as mean ± SEM. (Kaplan-Meier survival curves; Gehan-Breslow-Wilcoxon test).

Supplementary Figure S2: Representative Western blots of NMDA-NR1 in WT, SH and HK1 brain flies 2-3 days after enclosure.

Supplementary Figure S3: Original uncropped blots of replicates used for quantitative Western blot data in figure 6.
